# Early Changes in Microbial Colonization Selectively Modulate Intestinal Enzymes, but Not Inducible Heat Shock Proteins in Young Adult Swine

**DOI:** 10.1371/journal.pone.0087967

**Published:** 2014-02-04

**Authors:** Marie-Edith Arnal, Jing Zhang, Stefano Messori, Paolo Bosi, Hauke Smidt, Jean-Paul Lallès

**Affiliations:** 1 Food and Digestive, Central and Behavioral Adaptation Department, French National Institute for Research in Agriculture, Saint-Gilles, France; 2 Laboratory of Microbiology, Wageningen University, Wageningen, The Netherlands; 3 Department of Agricultural and Food Sciences, University of Bologna, Reggio Emilia, Italy; Louisiana State University and A & M College, United States of America

## Abstract

Metabolic diseases and obesity are developing worldwide in a context of plethoric intake of high energy diets. The intestine may play a pivotal role due to diet-induced alterations in microbiota composition and increased permeability to bacterial lipopolysaccharide inducing metabolic inflammation. Early programming of metabolic disorders appearing in later life is also suspected, but data on the intestine are lacking. Therefore, we hypothesized that early disturbances in microbial colonization have short- and long-lasting consequences on selected intestinal components including key digestive enzymes and protective inducible heat shock proteins (HSP). The hypothesis was tested in swine offspring born to control mothers (n = 12) or mothers treated with the antibiotic amoxicillin around parturition (n = 11), and slaughtered serially at 14, 28 and 42 days of age to assess short-term effects. To evaluate long-term consequences, young adult offspring from the same litters were offered a normal or a fat-enriched diet for 4 weeks between 140 and 169 days of age and were then slaughtered. Amoxicillin treatment transiently modified both mother and offspring microbiota. This was associated with early but transient reduction in ileal alkaline phosphatase, HSP70 (but not HSP27) and crypt depth, suggesting a milder or delayed intestinal response to bacteria in offspring born to antibiotic-treated mothers. More importantly, we disclosed long-term consequences of this treatment on jejunal alkaline phosphatase (reduced) and jejunal and ileal dipeptidylpeptidase IV (increased and decreased, respectively) of offspring born to antibiotic-treated dams. Significant interactions between early antibiotic treatment and later diet were observed for jejunal alkaline phosphatase and sucrase. By contrast, inducible HSPs were not affected. In conclusion, our data suggest that early changes in bacterial colonization not only modulate intestinal architecture and function transiently, but also exert site- and sometimes diet-specific long-term effects on key components of intestinal homeostasis.

## Introduction

Metabolic diseases, including insulin resistance, type 2-diabetes, obesity, hypertension and cardiovascular diseases are spreading worldwide, in a context of plethoric access to high energy-low fiber diets and limited physical activity [1]. Various tissues and organs are involved in these diseases. However, the intestine seems to play a pivotal role due to high fat (HF)-mediated increase in permeability to bacterial lipopolysaccharide (LPS) and LPS-induced metabolic inflammation [2]. The gut microbiota, the composition of which is sensitive to the diet appears crucial [2]. Even single bacteria (e.g. *Akkermansia muciniphila*) may control both intestinal and systemic outcomes as verified in mice [3].

The microbiota is now recognized as a vector of host development with respect to anatomy, physiology and metabolism [4]. In particular, neonatal bacterial colonization has been shown to impact gut angiogenesis, villus-crypt architecture, epithelial proliferation and apoptosis, and permeability [4]. Intestinal alkaline phosphatase (IAP) is a key enzyme in LPS detoxification and down-regulation of intestinal inflammation [5], [6]. Incidentally, IAP has been also considered as a heat shock protein (HSP)-like protein, due to its up-regulation upon heat stress [7]. Intestinal HSPs (HSP27 and HSP70) and enzymes like IAP are modulated by the microbiota [8], [9], [10]. HSP27 and HSP70 are induced following various kinds of stressors and are cytoprotective to the intestine [11], [12]. This could also be the case for intestinal HSP60 [13] although much less data are available. Both IAP and inducible HSP are defense systems highly conserved across evolution [12], [14].

Early programming of metabolic diseases appearing later in life was hypothesized three decades ago [15]. Since then, many tissues and organs have disclosed imprinted responses to nutritional or environmental disturbances *in utero* and (or) neonatally [16]. However, data on intestinal programming and long-term issues are still scarce [17]. We recently demonstrated in a rat model of intra-uterine growth retardation (IUGR) that IAP activity was imprinted as it increased in normal adult rats, but not in IUGR rats upon intake of a high fat (HF) diet [18]. As other intestinal enzymes (e.g. sucrase) and villus-crypt architecture were not influenced by IUGR itself or its interaction with adult diet in this model, we concluded that imprinting of intestinal function is a highly selective process. Work on early disturbances in neonatal gut colonization, e.g. by providing antibiotics to mothers or offspring revealed alterations in intestinal transcriptome and functional development [19], [20], but long term outcomes were not reported.

In the present study, we hypothesized that early changes in neonatal gut colonization have long-lasting consequences on selected intestinal functions, including protective HSPs and key enzymes involved in intestinal homeostasis. The hypothesis was tested in a swine model in which the mothers received a broad spectrum antibiotic orally around parturition, and offspring were serially sacrificed up to the age of 42 days (short-term study, ST), or submitted to a HF diet between 140 and 169 days and then slaughtered (long-term study, LT). The main outcome is that specific intestinal enzymes are selectively and site-specifically imprinted along the small intestine while inducible HSPs are not so in this swine model.

## Materials and Methods

### Experimental Procedure

Ethics statement: The experiment was designed and executed in 2010 in compliance with French and European law (Decree No. 2001–464 29/05/01, 86/609/CEE) for the care and use of laboratory animals. At that time (2010) getting approval from an ethic committee was not mandatory. One of us (JPL) held the authorization certificate No. 006708 for experimentation on living animals delivered by the French Veterinary Services. INRA Saint-Gilles, including the on-site slaughterhouse has an institutional license (agreement No. A35-622) from the French Veterinary Services.

Data relating to the present publication will be made available upon request.

All the animals of this experiment were reared in the conventional experimental premises of INRA Saint-Gilles (France) according to general rearing practices on site. Twenty four crossbred (Large White × Landrace) sows from our experimental herd, inseminated with Piétrain semen were used in two successive batches, taking into account parity and resistance of selected fecal bacteria to amoxicillin. This was determined for three bacteria (*Escherichia coli, Campylobacter* sp. and *Enterococcus* sp.) according to specific and accredited procedures carried out by the public veterinary laboratory of Rennes, France (ISAE, Rennes, France). Sows with amoxicillin-sensitive bacteria were assigned to the antibiotic group in priority, the remaining sows being assigned to the control (CTL) group. Groups of sows were located into different rooms of the same farrowing unit, and specific measures (e.g. separate rearing and intervention materials, circulation between rooms after changing clothes, etc.) were put in place to minimize cross contaminations between rooms. Broad spectrum antibiotic amoxicillin (Vetrimoxin PO containing 10% amoxicillin; CEVA Santé Animale, Loudéac, France) was provided daily to the sows (40 mg/kg body weight, BW) orally together with their morning meal (2 kg/day) in order to ensure total intake of offered amoxicillin. They were fed the rest of their daily feed allowance without supplemental antibiotic afterwards. The amoxicillin dosage used here was intermediate between values found in the literature for pigs (20 mg/kg BW/day [21]) and rats (100 mg/kg BW/day [22]). Amoxicillin was distributed from 10 days before the estimated farrowing date till 21 days after farrowing. Parturition was not induced.

Litter size was adjusted within treatment groups at n = 12 piglets per litter at the end of farrowing. Males were not castrated. Offspring were weighed at birth and then weekly until the end of the whole (ST and LT) experiment. Offspring were assigned to slaughter at the ages of 14, 28 (age of weaning), 42 (ST exp.) and 169 (LT exp.) days. Two days before each slaughter date in the ST experiment, offspring to be slaughtered (1 per litter) were selected from all litters so as to keep essentially similar means BW and BW variability between whole experimental groups and the sub-groups to be killed. Selected sub-groups were balanced for sex at each age of slaughter whenever possible. A similar selection process of offspring was made in the LT experiment, with the exception that homogeneous pairs of males or females were taken within litters and randomly assigned to either low (LF) or HF diet (see below) starting at the age of 140 days and lasting until day 169 (n = 10 litters per treatment). This experimental design allowed us to test the effect of diet within litters.

Sows and offspring were fed balanced diets formulated to cover nutritional requirements for gestating and lactating sows, and for starting (pre-starter and starter) and growing pigs [23] (Table S1, supplemental material). Sows were fed the gestating diet (3.5 kg/day) or the lactating diet (*ad libitum*) in two meals. Offspring had *ad libitum* access to all the feed formulas successively offered to them. The periods of feeding were as follows: pre-starter diet from weaning at 28 days till day 42, starter diet from day 43 till day 56; and growing diet (called LF) from day 57 till day 169. However, between day 140 and 169, a HF diet was obtained by adding palm oil (90 g/kg feed) to the LF diet. During this period, the pigs were reared in individual pens (2.25 m×0.85 m = 1.91 m^2^) and individual feed intake was measured. Finally, all the animals had free access to water.

### Collection of Feces in Sows

Sows’ feces were collected in sterile vials 4 weeks before theoretical farrowing date for determining amoxicillin sensitivity of selected bacteria. Feces were kept at +4°C during collection and then deposited within 2–3 hours at the veterinary laboratory (ISAE, Rennes, France). Sows’ feces were also collected at the initiation of antibiotic treatment and after 21 and 28 days of lactation for determining fecal microbiota composition. The samples were stored at −20°C and sent to the laboratory of Microbiology, Wageningen University (Wageningen, The Netherlands) on dry ice at the completion of each experimental (ST, LT) part.

### Animal Slaughter, and Digesta and Tissue Sample Collection

At the time points of interest, offspring were killed on site in our on-site EU-labelled slaughterhouse by electronarcosis immediately followed by exsanguination. A sample of blood was collected for analyzing acute phase proteins of inflammation in plasma. After laparotomy, 20-cm segments of proximal jejunum [beginning 10 cm (pigs aged 42 days or less) to 20 cm (pigs aged 169 days) distal to the ligament of Treitz] and distal ileum [beginning 5 cm (pigs aged 42 days or less) to 10 cm (pigs aged 169 days) proximal to the ileo-cecal valvula] was removed. Digesta were collected and frozen at −20°C, and empty tissue segments then flushed with sterile cold saline. Cross-sectional tissue samples were made for the following preparations or analysis: 5 cm for fixation (in buffered formalin 10%) paraffin embedding and histology of villi and crypts [18], [24]; 1 cm of whole tissue (cut in 3–4 pieces) for HSP analysis and another one stored in RNALater (Ambion, Austin, TX) 24 hours at +4°C and then storage at −20°C until mRNA extraction. The rest of collected segments was scraped using a glass slide for mucosal enzyme determination (rough homogenization, snap-freezing in liquid nitrogen and then storage at –20°C).

### Microbiota Analysis

Five sows and their offspring in each treatment were randomly selected in the first batch of pigs for microbial analysis in order to characterize our pig model. Microbial composition of fecal samples of sows collected at both beginning and end of antibiotic treatment (ATB) were analyzed using the Porcine Intestinal Tract Chip (PITChip), a phylogenetic microarray targeting the 16S ribosomal RNA genes of 627 porcine intestinal microbial species-level phylotypes [25], [26]. Accordingly, microbial composition of ileal and colonic content of their offspring collected at day 14, 21, 28 and 42 were analyzed by PITChip. Resulting images were processed using Agilent's Feature Extraction Software version 9.1 and further processed in R (library ‘microbiome ’ available from: http://microbiome.github.com).

### Villous and Crypt Morphometry

Intestinal tissue sections were prepared and characteristics of villi and crypts were measured as reported previously [18], [24]. Intestinal full size, well-oriented villi and crypts (10–15 per section) were measured for their length, width and surface area. Intestinal villus height-to-crypt depth ratio, and ‘M’ ratio for estimating three-dimension mucosal surface area [27] were calculated. Morphology parameters were averaged per animal prior to statistical analysis.

### Digestive Enzyme Activity Determination

The activities of alkaline phosphatase (IAP; E.C. 3.1.3.1), dipeptidyl-peptidase IV (DPP4; E.C. 3.4.14.5), aminopeptidase N (APN; E.C. 3.4.11.2) and sucrase (E.C. 3.2.1.48) were determined in intestinal mucosa homogenates as previously reported [18], [24]. Enzymes activities (or concentrations for IAP) were finally expressed per g of mucosa.

### Heat Shock Proteins and Heat Shock Factor-1

Soluble proteins from intestinal tissues were obtained as follows: frozen tissues were ground in liquid nitrogen and then extracted in borate buffer and protease inhibitor cocktail [28]. Proteins were assayed using Pierce™ BCA protein assay kit (ref. 23225; Thermo Scientific, Rockford, IL). Tissue HSP relative concentrations using β-actin as the reference protein were assayed by Western blotting as previously reported [28]. The equipment used for these analyses was new, and was applied together with commercially available gels, membranes and reagents (Bio-Rad, Marne-La-Coquette, France). They included: Mini Protean Tetra Cell system (ref. 165-8005) as the electrophoresis system, pre-casted 12% TGX gels (ref. 4561043) for protein migration and Trans Blot Turbo Transfer Starter System (ref. 170-4155) for protein transfer on PVDF membranes (ref. 170-4156). Molecular weight standards were also from Bio-Rad (ref. 161-10393). Ten micrograms of sample protein were deposited in each well and the electrophoresis was conducted at 160 V in a Tris/Glycine/SDS buffer (ref. 161-0772). Protein transfer was conducted at 2.5 A and 25V for 7 min in the Trans blot apparatus. Membranes were then blocked for 1 hour at room temperature in defatted milk powder prepared (50 g/L, ref. 170-6404) in Tris buffer saline (ref. 170-6435) and 0.1% Tween 20 (ref. 170-6531). The membranes were then incubated for 3 hours with primary antibodies prepared in the same mixture as for blocking. Except the anti-actin antibody that was from Sigma-Aldrich (ref. A2066), all the primary antibodies used were from Stressgen (Victoria, British Columbia, Canada), were produced in rabbits (except anti-HSP60 that was produced in goat) and were sold by Enzo Life Sciences (Villeurbanne, France): references SPA -803, -828-J, -812, -816, and -901 for anti-HSP27, -HSP60, -HSP70, -HSC70 and -HSF-1, respectively. Membranes were then washed 3×15 min in TBS Tween buffer before being incubated for 1 hour at room temperature with the second antibody coupled to horseradish peroxidase (ref. NA934, GE Healthcare Amersham for all the primary antibodies, except anti-HSP60 that was incubated with an anti-goat antibody ref. G50007 from InVitrogen, Camarillo, CA). After a final 3×15 min washing, protein bands on the membranes were stained by chemiluminescence using ECL-Plus reagent (ref. RPN2132, GE Healthcare Amersham) (2 ml per membrane, 5 min in the dark). Revealed bands were analyzed using an imager (ImageQuant™ LAS 4000, GE Healthcare). Protein band density was determined using UN-SCAN-IT gel 6.1 (Silk Scientific Inc., Orem, UT) and results of a given HSP band were expressed as a ratio to β-actin band density as determined for the same sample on the same membrane.

### qPCR Analysis

Intestinal mRNA was extracted, cDNA prepared and qPCR analysis performed as previously described [29]. Primers specific for intestinal enzymes and HSP were used [30]–[34] (Table S2, supplemental material).

### Blood Plasma α-acid Glycoprotein and Haptoglobin

Blood plasma α-acid glycoprotein (AGP) was measured using a commercial kit based on a radial immuno-diffusion assay according to the manufacturer's instructions (Cardiotech, Spring Lake, NJ, USA). Haptoglobin was determined by colorimetry using a commercial kit (Tridelta Ltd, Maynooth, Co. Kildare, Ireland) [35].

### Statistical Analysis

To relate changes in total bacterial community composition to treatment and sampling time (ST) or diet composition (LT), redundancy analysis (RDA) and Principal response curves were used as implemented in the CANOCO 4.5 software package (Biometris, Wageningen, the Netherlands). RDA is the canonical form of principle component analysis and is a multivariate linear regression method where several response parameters are related to the same set of environmental (explanatory) variables. The signal intensities for 144 genus-level phylogenetic groups of PITChip were used as responsive variables. Partial RDA was employed to analyze the effect of antibiotic treatment of sows on microbiota.

Statistical analysis of other data was carried out using the Statistical Analyzing System (SAS, version 8.1, 2000; SAS Institute Inc., Cary, NC, USA). Farrowing data were analyzed by GLM procedure for comparing CTL and ATB treatments. Offspring data were analyzed using MIXED models for testing the effects of treatment (against an error calculated between litters) and time of slaughter (error within litters) for the ST experiment, and the effects of treatment (between litters) and diet (within litters) for the LT experiment, respectively. The models also included the interaction term between early treatment and age of slaughter (ST exp.) or late diet (LT exp.). Results are presented as least-squares means and pooled SEM. Least-squares means comparisons for each combination of treatment and time were made only when a tendency (P≤0.10) for an interaction between these terms was observed. In these cases, means were separated using Bonferroni *post-hoc* comparisons. Data displaying a non-Gaussian distribution were log-transformed before statistical analysis. Effects were considered significant at P≤0.05 and as a trend at P≤0.10. Perinatal ATB treatment by age (ST exp.) or diet (LT exp.) and interaction (but not age effects *per se*) are commented.

Correlation of offspring ileal microbiota and enzymes (DPP-IV, PAI and APN) was analyzed by RDA in CANOCO 4.5. Treatments (control and antibiotic) were defined as nominal variables, whereas Age, DPP-IV, PAI and APN were defined as non-nominal variables. An interaction of age and treatments (control and antibiotic) was defined. Monte Carlo permutation test was used for assessing the significance of the contribution of environmental variables to the observed microbial variation.

## Results

### Sows’ Reproduction Performance

One sow in the ATB group died for unknown reasons two days after farrowing and its litter was excluded from the experiment. Antibiotic treatment of sows had no significant impact on reproduction performance. In particular, litter weight at birth amounted to 21.9 (1.4) and 21.0 (1.4) kg in CTL and ATB groups, respectively (P = 0.65). Pig numbers per litter at birth were not different between CTL and ATB groups [16.1 (1.03) and 14.1 (1.08), respectively; P = 0.19]. Average offspring birth weight was 1.38 (0.07) and 1.53 (0.07) kg in CTL and ATB groups, respectively (P = 0.12).

### Short-term Experiment

#### Zootechnical data and plasma proteins of inflammation

Offspring grew equally well between birth and day 42 and blood plasma analysis did not reveal significant treatment effects or interactions for AGP and haptoglobin (Table 1).

**Table 1:** Zootechnical data and plasma α-acid glycoprotein and haptoglobin concentrations in pigs born to control or antibiotics-treated sows and slaughtered at different ages (LSmeans and SEM, n = 9–12 per treatment and age).

*Sow’s treatment*	Control			Antibiotics				Statistics (P = )^1^		
*Offspring’s age*	d14	d21	d28	d14	d21	d28	SEM	treat.	diet	treat.*diet
**Performance**										
Birth BW (kg)	1.46	1.52	1.43	1.64	1.66	1.62	0.08	0.15	0.30	0.72
Slaughter BW (kg)	4.5	8.7	14.2	4.5	8.3	13.3	0.8	0.34	<0.0001	0.63
Daily BW gain (g)	222	254	286	216	253	283	15	0.86	0.0004	0.98
**Plasma proteins of inflammation**										
α-Acid glycoprotein (µg/mL)	905	722	994	839	756	950	54	0.58	0.001	0.63
Haptoglobin (µg/mL)	579	896	1038	760	234	1262	386	0.79	0.29	0.43

1 treat.: Treatment of sows pre- and post-partum (control versus antibiotics); diet (low versus high fat diet); treat.*diet: treatment by diet interaction.

#### Microbiota composition in sows and their offspring

**Figure 1:**
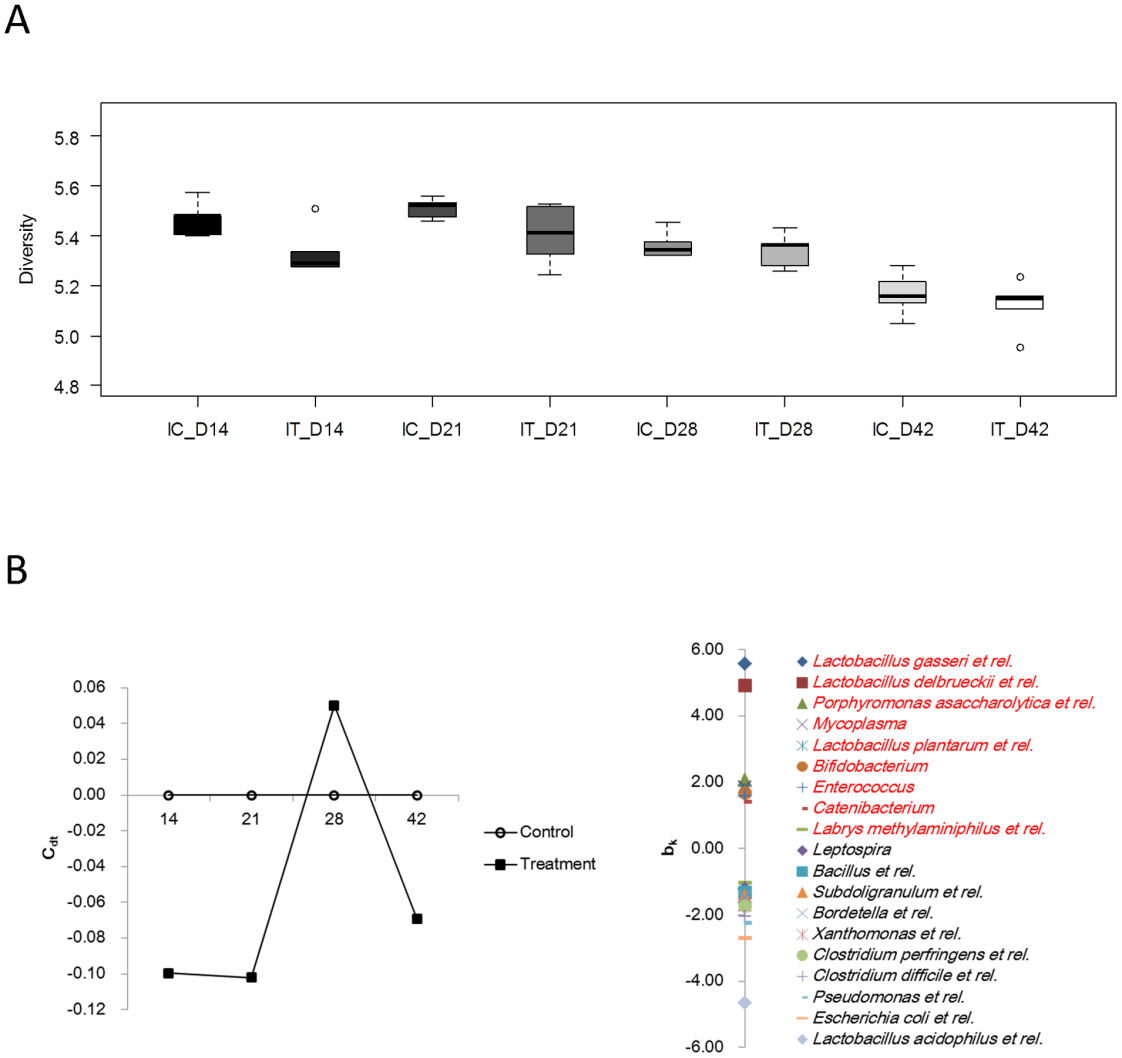
A. Diversity of offspring ileal microbiota expressed using the Shannon diversity index calculated based on probe-level signal intensities as implemented in the Microbiome package (http://microbiome.github.com). Maternal antibiotic treatment transiently reduced diversity on day 14 of age (P<0.05). B. Composition of offspring ileal microbiota was affected during the first weeks of life by maternal treatment, as evaluated by Principle Response Curve analysis of approximate genus-level microbial groups.

In order to characterize the model described here, and to show that antibiotic treatment of sows had repercussion on gut microbiota in both the sows and their offspring, we analyzed the microbial composition in sows’ feces as well as in intestinal contents of offspring for a subset of animals using the PITChip phylogenetic microarray. A more detailed description of microbial data will be published separately. ATB treatment of the sows transiently reduced microbial diversity in the ileum of piglets on day 14 after birth (Figure 1). Diversity in the colon was not affected at any time point (data not shown). Furthermore, composition of ileal microbiota was affected by the maternal ATB treatment, leading to a reduction in the relative abundance of several presumed beneficial bacterial populations such as lactobacilli and bifidobacteria, whereas increased relative abundance of potential pathogenic bacteria, including *Clostridium difficile, C. perfringens* and *E. coli*, was associated with the treatment group before day 21 (Figure 1). A similar trend was observed for colonic microbiota (data not shown).

Fecal microbiota composition of sows was affected by ATB treatment, leading to a similar reduction in relative abundance of lactobacilli as was also observed for the offspring (data not shown).

#### Intestinal architecture

In the jejunum, crypts tended to be wider (P = 0.098), and the M absorption factor lower (P = 0.054) in offspring born to ATB sows (Table 2). However, the treatment by age interaction was significant for these variables (P<0.10), showing wider crypts at day 14 and day 28 (but not at day 42) (P<0.05) and lower M absorption factor at day 14 (P<0.05) in ATB offspring. In the ileum, there was no significant ATB treatment effect, but interactions were observed for crypt depth and surface area (P<0.10). Crypt depth tended to be lower in ATB offspring at day 42 (P = 0.067) while differences between treatment groups for crypt surface area at a given age did not reach significance.

**Table 2:** Morphology of the jejunum of pigs born from control or antibiotics-treated sows and slaughtered at different ages (LSmeans and SEM, n = 9–12 per treatment and age).

*Sow’s treatment*	Control			Antibiotics				Statistics (P = )^1^		
*Offspring’s age*	d14	d21	d28	d14	d21	d28	SEM	treat.	diet	treat.*diet
**Jejunum**										
Villous height (VH, µm)	556	427	421	492	390	453	24	0.27	<0.0001	0.13
Villous width (µm)	112	147	161	115	143	163	4.6	0.96	<0.0001	0.75
Villous surface area (µm^2^,×10^3^)	55.2	52.7	60.9	48.9	47.9	64.3	2.9	0.31	0.0004	0.22
Crypt depth (CD, µm)	194	229	403	173	277	374	26	0.97	<0.0001	0.29
Crypt width (µm)	35.6^c^	42.7^b^	52.0^a^	40.2^b^	48.0^a^	49.4^a^	1.7	0.098	<0.0001	0.051
Crypt surface area (µm^2^×10^3^)	6.6	8.8	18.6	6.6	12.6	17.0	1.3	0.49	<0.0001	0.11
VH: CD ratio	2.94	2.04	1.34	3.01	1.76	1.67	0.19	0.82	<0.0001	0.32
Absorption surface magnif. ‘M’	12.1^a^	7.6^c^	6.3^c^	10.0^b^	6.4^c^	6.9^c^	0.5	0.054	<0.0001	0.057
**Ileum**										
Villous height (VH, µm)	528	280	354	507	338	348	34	0.74	<0.0001	0.48
Villous width (µm)	113	106	151	109	115	145	4.5	0.98	<0.0001	0.16
Villous surface area (µm^2^,×10^3^)	50.0	25.3	46.5	48.3	33.0	41.6	4.3	0.93	0.0002	0.33
Crypt depth (CD, µm)^2^	124^b^	119^b^	189^a^	135^b^	121^b^	169^a^	7	0.72	<0.0001	0.092
Crypt width (µm)	44.6	43.1	56.3	44.3	45.8	53.9	1.6	0.99	<0.0001	0.28
Crypt surface area (µm^2^×10^3^)	4.88^c^	4.53^c^	9.19^a^	5.39^c^	4.84^c^	7.76^b^	0.45	0.59	<0.0001	0.068
VH: CD ratio	4.34	2.39	1.92	3.90	2.79	2.15	0.28	0.79	<0.0001	0.30
Absorption surface magnif. ‘M’	10.2	5.8	5.4	9.7	6.6	5.8	0.5	0.64	<0.0001	0.52

1 treat.: Treatment of sows pre- and post-partum (control versus antibiotics); diet (low versus high fat diet); treat.*diet: treatment by diet interaction.

2 Crypt depth: tended to be lower in ATB than CTL group at day 42 (*P = 0.067*).

a,b,c, Means within rows with different letters differ (P<0.05).

#### Mucosal enzymes

**Figure 2:**
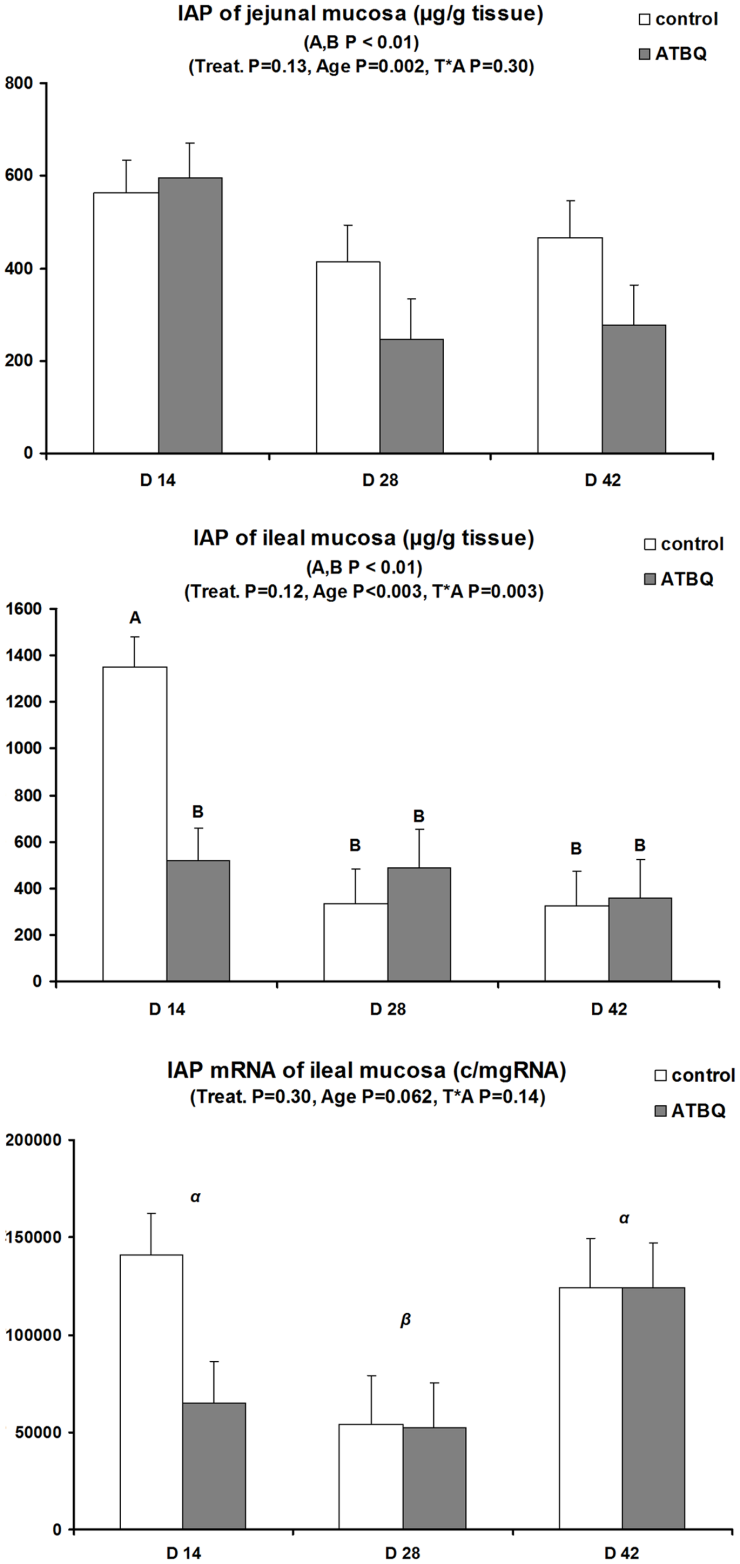
Intestinal alkaline phosphatase (IAP) concentration in jejunal and ileal mucosae and IAP mRNA expression in ileal tissue of offspring born to control or antibiotic-treated sows and slaughtered at different ages (LSmeans and SEM, n = 9–12 per treatment and age). Jejunal IAP was not influenced by treatments, but ileal IAP was transiently lower at day 14 (P<0.05) in offspring born to antibiotic-treated sows compared to controls (treatment by time interaction, P = 0.003). Differences in Ileal IAP mRNA levels did not reach significance, but ileal IAP concentrations and mRNA levels were positively correlated (P<0.01).

Jejunal enzyme activities were not influenced by the factors tested (Table 3). By contrast, significant interactions between treatment and age were observed for ileal APN and DPP-IV (P = 0.070 and P = 0.047, respectively). Indeed, differential age effects between ATB and CTL offspring were seen for APN, while DPP-IV activity was higher in ATB offspring than in controls at day 28 (P<0.05). Jejunal IAP activity did not vary significantly according to treatments (Figure 2). Contrasting with this, there was a highly significant interaction between ATB treatment and age for ileal IAP activity (P = 0.003) (Figure 2). IAP activity was nearly three-fold lower in ATB offspring at day 14 (P<0.01), with no treatment differences thereafter. Although effects of tested factors did not reach significance for ileal IAP mRNA levels, positive correlations (P<0.01) were observed between IAP activity and mRNA levels.

**Table 3:** Total Activities^1^ of aminopeptidase N, dipeptidyl-peptidase IV and sucrase in the jejunum and ileum of pigs born to control or antibiotics-treated sows and slaughtered at different ages (LSmeans and SEM, n = 9–12 per treatment and age).

*Sow’s treatment*	Control			Antibiotics				Statistics (P = )^2^		
*Offspring’s age*	d14	d21	d28	d14	d21	d28	SEM	treat.	diet	treat.*diet
**Jejunum**										
Aminopeptidase N (APN)	10.1	10.9	10.3	9.4	8.4	8.1	1.4	0.13	0.92	0.75
Dipeptidyl-peptidase IV (DPP-IV)	0.82	0.80	0.73	0.71	0.77	1.05	0.11	0.53	0.48	0.13
Sucrase	2.9	3.2	1.2	3.0	3.0	1.6	0.6	0.83	0.014	0.88
**Ileum**										
Aminopeptidase N (APN)	8.3^b^	9.8^ab^	12.6^a^	6.1^b^	11.9^a^	10.2^ab^	1.1	0.38	0.0006	0.070
Dipeptidyl-peptidase IV (DPP-IV)	3.65^a^	1.78^b^	1.89^b^	2.77^ab^	3.28^a^	2.37^ab^	0.47	0.37	0.075	0.047
Sucrase (log)	−0.09	0.47	0.62	−0.41	0.50	0.55	0.11	0.20	<0.0001	0.027

1 Total activity (µmoles/min/g mucosa); × 10^−3^ for DPP-IV.

2 treat.: Treatment of sows pre- and post-partum (control versus antibiotics); age (d14 and d28, unweaned; d42 weaned at d28); treat.*age: treatment by age interaction.

a,b Means within rows with different letters differ (P<0.05).

#### Heat shock proteins and heat shock factor-1

**Figure 3:**
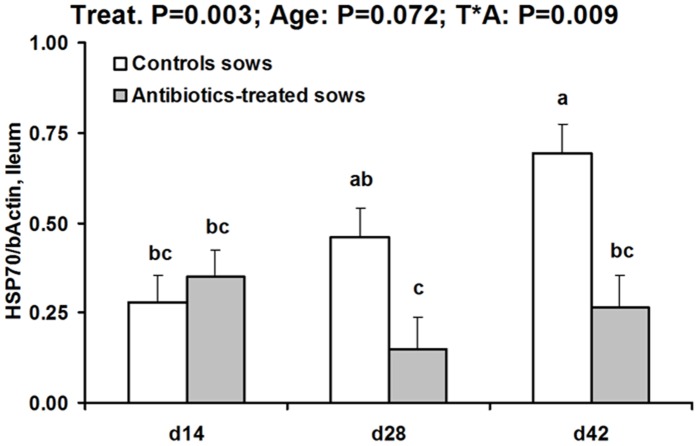
Heat shock protein 70 concentration (relative to β-actin) in ileal tissue of offspring born to control or antibiotic-treated sows and slaughtered at different ages (LSmeans and SEM, n = 9–12 per treatment and age). Ileal HSP70 relative concentration was not different between groups at day 14, but it was significantly lower (P<0.05) at day 28 and day 42 in offspring born to antibiotic-treated sows compared to controls (treatment by time interaction, P = 0.009).

Jejunal HSP70 (but not HSP27) was influenced by ATB treatment of sows (global effect −20%, P = 0.031) (Table 4). Offspring ileal HSP27 was not influenced by the tested factors. By contrast, ileal HSP70 was lower in ATB than CTL offspring (P = 0.003) and there was a significant treatment by age interaction (P = 0.017) (Figure 3). While there was not treatment effect at day 14, ileal HSP70 protein level was two- to three-fold lower in ATB than CTL offspring at day 28 and day 42 (P<0.05). Ileal tissue expression levels for HSP27 and HSP70 were not significantly influenced by the tested factors (Table S4). Ileal HSC70 protein level was higher in ATB than CTL offspring (P = 0.05). This effect precluded the use of HSC70 as a reference protein for inducible HSPs. HSP60 in the ileum (not assayed in the jejunum) tended to be higher in ATB than CTL offspring (P = 0.070). HSF-1, the heat shock factor regulating inducible HSP expression was not influenced in the ileum by the factors tested (Table 4).

**Table 4:** Protein expression of heat shock proteins and heat shock factor-1 in intestinal tissues of pigs born to control or antibiotics-treated sows and slaughtered at different ages (LSmeans and SEM, n = 9–12 per treatment and age).

Sow’s treatment	Control			Antibiotics				Statistics (P = )^1^		
Offspring’s age	d14	d21	d28	d14	d21	d28	SEM	treat.	diet	treat.*diet
**Jejunum**										
HSP27/b-Actin	0.97	1.08	1.20	0.89	0.91	1.02	0.12	0.17	0.30	0.90
HSP70/b-Actin	0.61	0.74	0.81	0.52	0.59	0.63	0.07	0.031	0.12	0.84
**Ileum**										
HSP27/b-Actin	0.77	0.38	0.82	0.53	0.68	0.61	0.15	0.69	0.46	0.12
HSC70/b-Actin	0.82	0.76	0.75	1.04	1.12	0.73	0.11	0.050	0.11	0.23
HSP60/b-Actin	0.62	0.31	0.54	0.70	0.64	0.72	0.12	0.07	0.25	0.58
HSF1/b-Actin	0.33	0.38	0.41	0.33	0.31	0.41	0.08	0.69	0.51	0.87

1 treat.: Treatment of sows pre- and post-partum (control versus antibiotics); diet (low versus high fat diet); treat.*diet: treatment by diet interaction.

#### Correlations between microbiota composition and intestinal parameters

**Figure 4:**
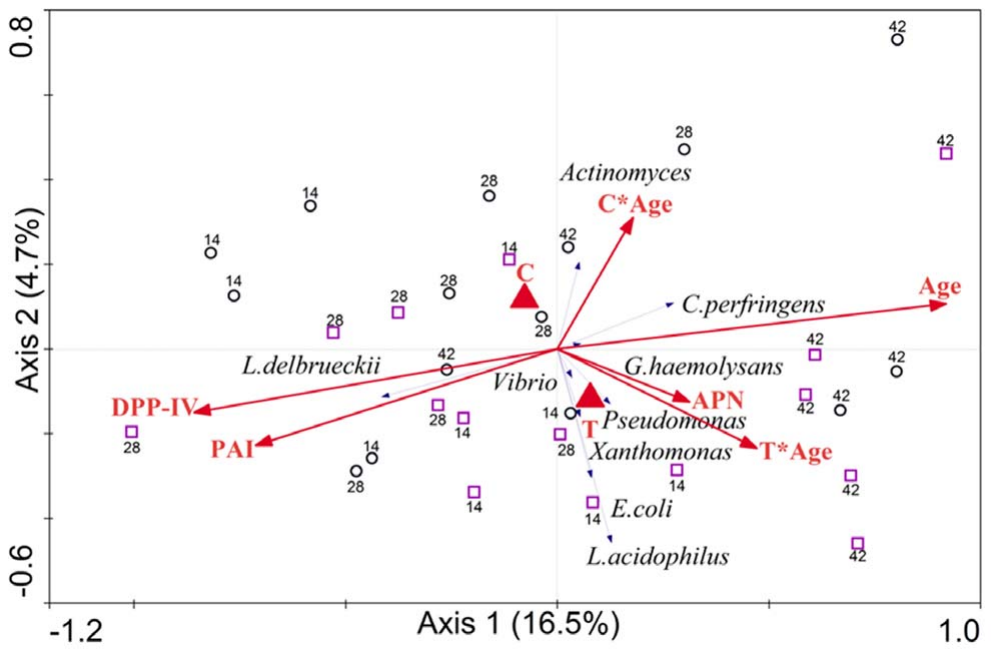
Correlations between microbiota composition and intestinal parameters, using RDA as implemented in CANOCO 4.5. Treatments (control and antibiotic) were defined as nominal variables, whereas Age, DPP-IV, PAI and APN were defined as non-nominal variables. An interaction of age and treatments (control, C*Age, and antibiotic, T*Age) was defined. Monte Carlo permutation test was used for assessing the significance of the contribution of environmental variables to the observed microbial variation.

Correlation analysis between microbiota composition and intestinal parameters revealed that IAP and DPP4 activities were positively correlated with *L. delbrueckii* and negatively correlated with *C. perfringens* (Figure 4). The activity of APN was correlated positively with C. *perfringens, E. coli* and *L. acidophilus,* and negatively correlated with *L. delbrueckii* (Figure 4). Furthermore, age was found to significantly contribute to the microbial variation (P = 0.004) and DPP-IV tended to contribute significantly (P = 0.084).

### Long-term Experiment

#### Zootechnical data and plasma proteins of inflammation

There were no differences in body weight between treatment groups at the beginning and at the end of the LT trial, so that growth rates were essentially similar (Table 5). The level of intake of feed that were offered *ad libitum* was 10% lower in HF offspring (P<0.05), but the level of metabolizable energy intake did not differ between treatment groups. Plasma AGP concentration was 26% higher in offspring born to ATB sows (P<0.05) while haptoglobin concentration tended to be higher in HF pigs (+29%, P = 0.070) compared to LF pigs.

**Table 5:** Zootechnical data and plasma concentrations of α-acid glycoprotein and haptoglobin in offpsring born to control or antibiotics-treated sows and fed a low (LF) or high (HF) fat diet between 140 and 169 days of age (LSmeans and SEM, n = 10 per treatment).

*Sow’s treatment*	Control		Antibiotics			Statistics (P = )^1^		
*Offspring’s diet*	LF	HF	LF	HF	SEM	treat.	diet	treat.*diet
**Performance**								
BW, day 140 (kg)	93.2	92.0	93.0	88.8	1.1	0.14	0.32	0.15
BW, day 169 (kg)	127.8	124.9	128.4	125.3	1.5	0.79	0.38	0.93
ADG, day 140-day 169 (kg)	1.21	1.15	1.21	1.25	0.04	0.26	0.88	0.19
ADFI day 140-day 169 (g/kg BW)^2^	27.6	24.3	26.6	24.6	0.7	0.65	0.046	0.31
ADMEI day 140-day 169 (KJ/kg BW)^3^	347	349	334	354	11	0.66	0.29	0.31
**Plasma proteins of inflammation**								
α-Acid glycoprotein (µg/mL)	357	344	421	465	37	0.028	0.67	0.45
Haptoglobin (µg/mL)	1650	2219	1906	2359	265	0.47	0.070	0.83

1 treat.: Treatment of sows pre- and post-partum (control versus antibiotics); diet (low versus high fat diet); treat.*diet: treatment by diet interaction.

2 BW: body weight. ADFI: average daily feed intake: 27.1 (0.5) and 24.4 (0.5) g/kg BW for pigs fed the LF and HF diets, respectively (P = 0.046).

3 ADMEI: Average daily metabolizable energy intake.

#### Microbiota composition in 6 month-old offspring

In contrast to the observations made during the first 6 weeks of life in piglets, no significant effects of ATB on microbiota was observed at the long term, even though there was a tendency for an interaction between ATB treatment and offspring adult diet (P = 0.065, data not shown).

#### Intestinal architecture

Intestinal architecture of pigs in the LT trial was very little influenced by treatments as the only observed difference was for crypts that were slightly (+6%) wider in ATB offspring compared to CTL (P<0.05) (Table S3, Supplemental material).

#### Mucosal enzymes

**Figure 5:**
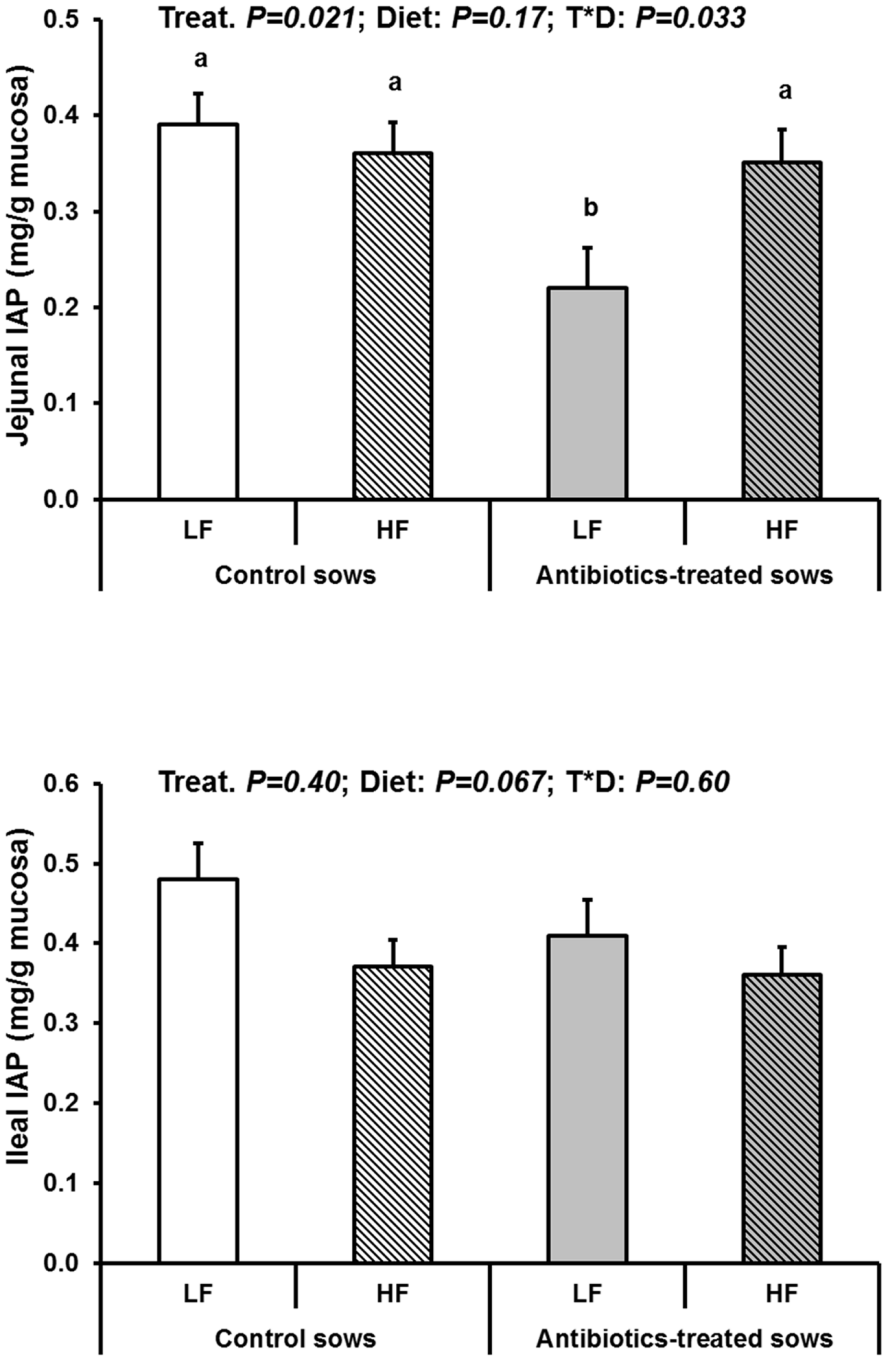
Intestinal alkaline phosphatase (IAP) concentration in jejunal and ileal mucosae in jejunal tissue of offspring born to control or antibiotic-treated sows and fed a LF or a HF diet between 140 and 169 days of age (LSmeans and SEM, n = 10 per treatment and diet). Jejunal IAP was significantly lower (P<0.05) in jejunal mucosa of offspring born to antibiotic-treated sows compared to controls when fed the LF diet (treatment by diet interaction, P = 0.03). The effects of the tested factors were not significant for ileal IAP concentration or mRNA levels.

Jejunal DPP-IV activity was 86% higher in ATB offspring than in CTL (P<0.01) (Table 6). Jejunal sucrase was on average 20% lower in HF compared to LF offspring (P = 0.020). In fact, there was an interaction between ATB treatment and diet composition for this enzyme (P = 0.047). Sucrase activity was lower in CTL offspring fed the HF diet compared to those fed the LF diet (P<0.05) while it was not influenced by the diet in the ATB offspring. Opposed with observations in the jejunum, ileal DPP-IV activity was lower in ATB offspring compared to CTL (−32%, P = 0.028) while ileal sucrase activity was not influenced by the tested factors. Jejunal and ileal APN activities were not influenced, but jejunal IAP activity was 24% lower in ATB compared to CTL offspring (P = 0.021) (Figure 5). There was also a treatment by diet interaction for this enzyme (P = 0.033). ATB offspring displayed a nearly halved jejunal IAP activity (P<0.05) compared to CTL offspring with the LF diet while there was no difference between ATB and CTL with the HF diet. Ileal IAP displayed only a trend (P = 0.067) for lower activity in HF compared to LF offspring. Finally, in terms of gene expression, only sucrase mRNA relative levels displayed a treatment by diet interaction (P = 0.013) (Table 7). Sucrase mRNA level was lower in ATB than in CTL offspring when fed the LF diet, with non-significant differences between treatments when fed the HF diet. However, gene expression data may have been biased by the fact that one fourth (11/40) of the samples displayed poor quality mRNA and where therefore discarded.

**Table 6:** Enzyme activities^1^ of jejunal and ileal mucosa in pigs born to control or antibiotics-treated sows and fed a low (LF) or high (HF) fat diet between 140 and 169 days of age (LSmeans and SEM, n = 10 per treatment).

*Sow’s treatment*	Control		Antibiotics			Statistics (P = )^1^		
*Offspring’s diet*	LF	HF	LF	HF	SEM	treat.	diet	treat.*diet
**Jejunal mucosa**								
Aminopeptidase N (APN)	6.8	6.1	6.1	7.2	0.6	0.80	0.69	0.14
Dipeptidyl-peptidase IV (DPP-IV)^3^	0.42	0.35	0.77	0.65	0.08	0.001	0.26	0.76
Sucrase^4^	3.8^a^	2.6^c^	3.0^b^	2.9^b^	0.3	0.37	0.020	0.047
**Ileal mucosa**								
Aminopeptidase N (APN)	12.4	10.7	11.1	9.9	1.2	0.41	0.23	0.84
Dipeptidyl-peptidase IV (DPP-IV)^5^	1.9	1.8	1.3	1.2	0.2	0.028	0.69	0.99
Sucrase	4.9	3.8	4.8	4.6	0.4	0.58	0.18	0.34

1 Total activity (µmoles/min/g mucosa) (nmoles/min/g mucosa for DPP-IV).

2 treat.: Treatment of sows pre- and post-partum (control versus antibiotics); diet (low versus high fat diet); treat.*diet: treatment by diet interaction.

3 Treatment effect for dipeptidyl-peptidase activity: 0.38 (0.06) and 0.71 (0.06) nmoles/min/g mucosa for controls and antibiotics, respectively (P = 0.001).

4 Diet effect for sucrase TA: 3.4 (0.2) and 2.7 (0.2) nmoles/min/g mucosa for LF and HF, respectively (P = 0.020).

5 Treatment effect for dipeptidyl-peptidase activity: 1.8 (0.2) and 1.2 (0.2) nmoles/min/g mucosa for controls and antibiotics, respectively (P = 0.028).

a,b,c Means with different superscript letters within rows differ (P<0.05).

**Table 7:** mRNA expression levels of housekeeper and digestive enzyme genes in jejunal tissue of pigs born to control or antibiotics-treated sows and fed a low (LF) or high (HF) fat diet in young adulthood (LSmeans and SEM, n = 5–9 per treatment^1^).

*Sow’s treatment*	Control		Antibiotics			Statistics (P = )^1^		
*Offspring’s diet*	LF	HF	LF	HF	SEM	treat.	diet	treat.*diet
Cyclophilin A	2.60	3.13	2.24	2.51	0.32	0.16	0.25	0.70
Aminopeptidase N/Cyclo A	0.68	0.42	0.54	0.50	0.10	0.76	0.16	0.33
DPPIV/Cyclo A^3^	nd	nd	nd	nd	nd			
Sucrase/Cyclo A^4^	2.03^a^	1.06^b^	1.23^b^	1.61^ab^	0.23	0.61	0.23	0.013
Alkaline phosphatase/Cyclo A	1.33	1.03	0.93	0.99	0.19	0.28	0.55	0.37

1 Due to poor quality of mRNA in 11 out of 40 samples.

2 treat.: Treatment of sows pre- and post-partum (control versus antibiotics); diet (low versus high fat diet); treat.*diet: treatment by diet interaction.

3 nd: not done because the primers used (Petersen et al. 2001) did not work.

4 Treatment by diet interaction (*P = 0.013*). CTL-LF different from CTL-HF (*P = 0.013*) and from ATBQ-LF (*P = 0.016*).

a,b Means with different superscript letters within rows differ (*P<0.05*).

#### Heat shock proteins and heat shock factor-1

Protein levels of HSPs and HSF-1 in intestinal tissues of pigs reared in the LT experiment were not influenced by early ATB treatment or composition of the growing diet (Table 8). Therefore, corresponding mRNA relative levels were not assessed.

**Table 8:** Protein expression of heat shock proteins and heat shock factor-1 in intestinal tissues of pigs born to control or antibiotics-treated sows and fed a low (LF) or high (HF) fat diet in young adulthood (LSmeans and SEM, n = 10 per treatment).

*Sow’s treatment*	Control		Antibiotics			Statistics (P = )^1^		
*Offspring’s diet*	LF	HF	LF	HF	SEM	treat.	diet	treat.*diet
**Jejunum**								
HSP27/b-Actin	0.56	1.06	0.85	0.78	0.20	0.96	0.28	0.15
HSP70/b-Actin	1.02	0.87	0.86	0.85	0.11	0.43	0.45	0.11
**Ileum**								
HSP27/b-Actin	0.99	1.13	1.00	1.07	0.10	0.81	0.31	0.70
HSP70/b-Actin	1.01	0.86	1.05	0.90	0.17	0.82	0.37	1.00
HSC70/b-Actin	0.66	0.48	0.55	0.64	0.10	0.80	0.65	0.18
HSF1/b-Actin	0.10	0.13	0.13	0.15	0.02	0.15	0.29	0.77

1 treat.: Treatment of sows pre- and post-partum (control versus antibiotics); diet (low versus high fat diet); treat.*diet: treatment by diet interaction.

## Discussion

We report important data on ST and LT effects of early changes in microbial colonization on small intestinal biology in a swine model of maternal antibiotic treatment around parturition. We show that this treatment transiently induced diverse temporal and regional patterns of selective modifications in crypt depth, IAP activity and HSP70 protein production that collectively suggest a lower or delayed host response to bacteria especially in the ileum. Importantly, LT investigations reveal region-specific and selective changes in particular enzymes (e.g. IAP, sucrase) while other protective components like inducible HSPs were not influenced in the long term in this model.

### The Pig Model: Effects of ATB Treatment on the Microbiota of Sows and Offspring

In line with changes in intestinal microbiota observed in this study, amoxicillin nearly eradicated lactobacilli in the small intestine of rat pups [19]. It is interesting to note that we found similar changes in the relative abundance of groups within the lactobacilli in the sows’ fecal microbiota as were observed for ileal and colonic microbiota of offspring during the first 6 weeks of life. This points towards an indirect effect of maternal ATB treatment via the sow’s own microbiota that is transmitted to their offspring during the initial colonization of the newborn piglet. By contrast, long-term effects on offspring gut microbiota composition appeared to be limited.

### Influences on small Intestinal Morphology, Enzymes and Inducible HSPs

#### Short-term influence of maternal ATB treatment on offspring small intestine

The consequences of maternal ATB treatment, although mild were more pronounced in the distal ileum as compared to the proximal jejunum. Regarding intestinal morphology, shorter crypt depth and smaller crypt surface area in ATB offspring were observed at 42 days of age. This would suggest an impaired or delayed host response to bacteria in the ileum in this group, mediated by the observed differences in microbiota diversity and composition early in life. To this end, it is interesting to note that conventional pigs display deeper crypts than germ-free or mono-colonized pigs [36]. However, villus architecture remained essentially unaffected in the present study. Elongated villi were observed in the ileum (but not in the proximal jejunum) of germ-free or mono-colonized piglets with a non-pathogenic *E. coli* strain [36]. When amoxicillin was given to weanling pigs for 21 or 27 days, no effects on villus or crypt architecture were noted [37] ), and it is tempting to speculate that treatment-mediated changes in microbiota composition significantly affect architecture very early during initial colonization, rather than later in life. Although calculated jejunal absorptive surface area (‘M’ factor) was lower at day 14 in ATB offspring, in association with reduced IAP activity, this had no apparent consequence on growth performance or on systemic inflammation during that period.

For intestinal enzymes, ileal IAP activity was transiently reduced in ATB offspring, suggesting also a lower threat possibly caused by less Gram-negative bacteria (or less bacterial pro-inflammatory components, e.g. LPS) on this segment. Indeed, conventionalization of zebrafish with Gram-negative (but not Gram-positive) bacteria increased IAP activity [9]. In pigs, reduced specific activity (/g protein) of IAP was reported after mono-association with non-pathogenic *E. coli* strains, but total activities were not reported [38] so that comparisons with our work are difficult. Finally, interactions between treatment and age for ileal APN and DPP-IV are difficult to interpret as they did not reveal very clear patterns of variation (Table 3). Conventional pigs were reported to display reduced APN activity compared to germ-free pigs, possibly as a consequence of APN protein degradation by enteric bacteria [39].

Little is known on the possible relationships between intestinal microbiota and digestive enzymes. Various *L. delbrueckii* strains display anti-inflammatory properties on intestinal epithelial cell lines in cultures [40]. This may contribute to explain the positive correlation seen here between *L. delbrueckii* and IAP as this enzyme is down-regulated by inflammation [5]. However, the cause-and-effect relationship cannot be determined from the present work because IAP is a potent anti-inflammatory component of the small intestine, and inflammation by itself impacts gut microbiota composition [5], [6]. A negative correlation was found between *C. perfringens* and IAP (Figure 4). Again, this may reflect the anti-inflammatory properties of IAP as anti-inflammatory substances or probiotic strains were able to reduce *C. perfringens*-induced intestinal lesions and to increase ileal IAP in chickens [41], [42]. To the best of our knowledge, no information is available thus far on putative relationships between bacteria correlating positively (e.g. *C. perfringens, E. coli and L. acidophilus*) or negatively (e.g. *L. delbrueckii*) with activities of enzymes like IAP, DPP4 or APN.

Luminal bacteria were responsible for physiological expression of protective HSP25 and HSP72 (corresponding to HSP27 and HSP70 in human) in the small intestine of rats, while treatment with the antibiotic metronidazole depressed intestinal HSP expression [43]. This is consistent with our present observation that offspring born to ATB-treated dams displayed reduced HSP70 protein levels in the ileum and jejunum at 28 and 42 days of age. HSP70 is a molecular chaperone protecting intestinal epithelial cell structure and function [11], [12]. The regulatory mechanism of HSP70 decrease was not transcriptional, and HSP70 response was not linked to changes in protein production of HSF-1, a key transcription factor involved in the initiation of the heat shock response [44]. Our data thus suggest a post-translational regulation of intracellular HSP70 concentration, a point which needs to be investigated deeper in future studies. Reduced HSP70 protein level further supports lower or delayed host response to bacteria in the small intestine of offspring born to ATB-treated sows. Intestinal HSP27 was not impacted in the present work. Comparisons between studies (e.g. [43]) are difficult because of differences in animal species, relative age and antibiotic used, but this may suggest a mild effect of dam ATB treatment on offspring intestine as only HSP70 was affected. Microbiota data could suggest that decreased HSP70 may be related to the depletion of various lactobacilli. This is because certain lactobacilli have been shown to stimulate inducible HSP expression [45], [46]. Conversely, increased *E. coli* relative counts in the ileum may probably not account for such HSP changes. Indeed, *E. coli* LPS was shown to stimulate only HSP25 protein production, but this was in cultured intestinal epithelial cells [47], and HSP27 expression was not influenced by the treatment in the present study. Ileal HSP60 tended to be higher in ATB offspring. Recent data with cultured intestinal epithelial cells suggest a protective role for this HSP against oxidative stress and inflammation [13], but much less is known on it, as compared to HSP27 and HSP70 *in vivo*. The decrease in ileal HSP70 associated with a trend for increased HSP60 may be suggestive of compensatory mechanisms within the HSP family, with final outcomes (e.g. sensitivity to oxidative or inflammatory stress) being difficult to predict.

Systemically, we did not find any evidence of differential inflammation between ATB and CTL offspring. This is in sharp contrast with data by Fak et al. [20] who reported twice higher plasma levels of haptoglobin in rats born to dams treated with an antibiotic mixture (metronidazole, neomycin, polymyxin B) compared to non-treated controls. As *in vivo* intestinal permeability was altered, aberrant intestinal colonization by *Enterobacteriaceae* (e.g. *E. coli*) in ATB offspring was suggested to be responsible for this inflammation [20]. Plasma AGP is an inflammatory protein [48] and recent data suggest AGP as a potential marker of growth impairment in newborn pigs [49]. The lack of difference in plasma AGP concentration between treatment groups is in agreement with similar growth patterns between groups observed here.

Collectively, the results from our ST experiment suggest a transiently disturbed or delayed host response to bacteria mainly in the distal small intestine of offspring born to ATB-treated mothers. This is suggested by transient reductions in IAP, inducible HSPs and crypt depth.

#### Long-term influence of maternal ATB treatment on offspring small intestine

The major finding of the present work is that early disturbances in bacterial colonization can have long-lasting effects on specific intestinal traits (e.g. some key intestinal enzymes) although bacterial diversity seemed to be little affected. In particular, we observed a two-fold reduction in jejunal IAP activity of ATB offspring. IAP is a key enzyme recently demonstrated to dephosphorylate and thus detoxify bacterial LPS [5], [6]. LPS is known to be pro-inflammatory, and anti-inflammatory properties of intestinal IAP both locally and systemically are well documented [5], [6]. Our data could indicate differential variations in LPS detoxification capacity along the small intestine of ATB offspring as compared to controls. Adult rats born IUGR showed early programming of jejunal IAP, but this was disclosed only under the HF diet [18]. Although our two studies differ in animal species and models of early disturbances, the common conclusion is the susceptibility of IAP to early influences as revealed in adulthood. This is an important finding because intestinal detoxification of LPS by IAP is a highly conserved function across evolution [14]. However, underlying mechanisms of IAP modulation warrant further investigation.

**Figure 6:**
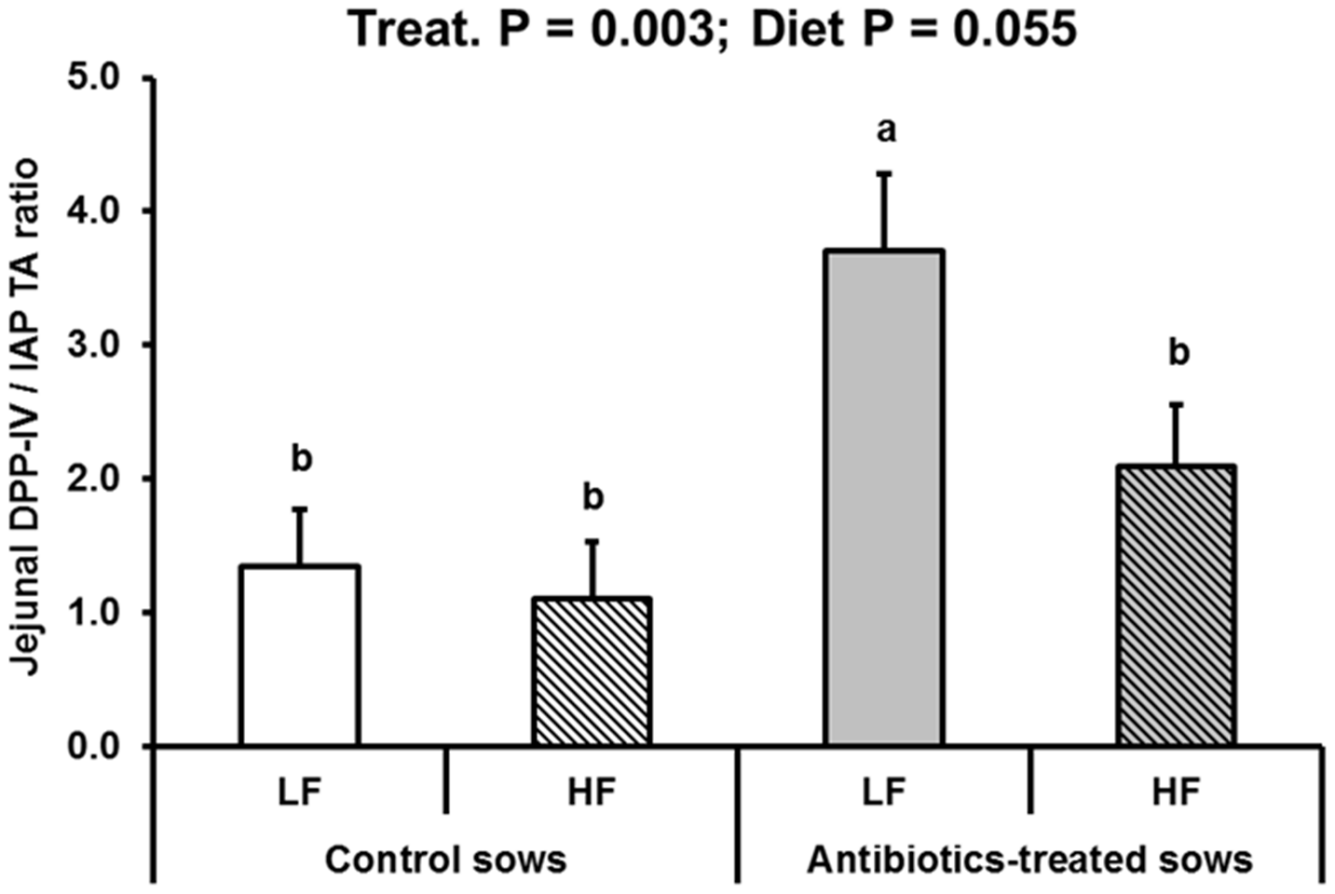
Dipeptidylpeptidase IV-to-intestinal alkaline phosphatase ratio in jejunal mucosa of offspring born to control or antibiotic-treated sows and fed a low fat (LF) or a high fat (HF) diet between 140 and 169 days of age (LSmeans and SEM, n = 10 per treatment and diet). Jejunal DPP-IV-to-IAP ratio was higher in offspring born to antibiotic-treated sows compared to controls (P = 0.003) and tended to be lower in offspring fed the HF compared to the LF diet (P = 0.055).

DPP-IV and APN peptidases have been investigated due to their putative broad pro-inflammatory role [47], [50]. More specifically, intestinal DPP-IV is causally involved in glucose intolerance in mice [51]. DPP-IV hydrolyzes the incretins glucagon-like peptide 1 and glucose-dependent insulinotropic peptide, a process that generates two bioactive dipeptides responsible for glucose tolerance deterioration and reduced insulin secretion [51]. Here, we found that DPP-IV activity responded differentially in the jejunum (ATB>CTL) and the ileum (ATB<CTL) (Table 3). However, it is difficult to conclude on the global outcomes of such effects because we did not make an assessment of total quantities of DPP-IV enzyme along the small intestine, and investigating plasma incretins was not our aim here. Anyhow, the major conclusion of the present observation is that intestinal DPP-IV is modulated in the long-term, although underlying mechanisms are still unclear. As high intestinal DPP-IV may be detrimental [51] and IAP protective [5], [6], we calculated the jejunal DPP-IV to IAP ratio (Figure 6) as an ‘intestinal risk index’ for metabolic disorders. We found this index to highly discriminate between ATB and CTL offspring, as it was so in adult rats born IUGR compared to controls [18]. Incidentally, plasma AGP was also higher in adult offspring born to ATB sows, suggesting some form of systemic inflammation in these pigs. However, the actual function of AGP, which does not correlate with haptoglobin remains obscure [48], [49].

Sucrase has long been considered as a maturation marker of intestinal epithelial cells [52]. In the present study, jejunal (but not ileal) sucrase activity and mRNA levels were influenced by adult diet composition in CTL, but not in ATB offspring, suggesting an imprinting. This is in contrast with our rat study where jejunal sucrase was not influenced [18], highlight species-specific differences.

Finally, inducible HSP27 and HSP70 (and possibly HSP60) have been reported many times to be protective against oxidative stress and inflammation of the intestine [11], [12]. In the present study, the investigated intestinal HSPs did not appear to be influenced in the long-term.

#### Influence of the diet in adulthood

Although testing dietary influences *per se* in our LT study was not our aim, it is a useful tool for investigating how early life events may interact with the diet, and especially unbalanced diets (like HF) at risk for metabolic diseases and obesity in later life. Here, effects of the HF diet were rather limited contrary to rodent experiments where larger amounts of fatty diets (fat providing between 30 and 60% of energy intake) are usually offered (e.g. [2], [18]). No differences in energy intake or in growth rate were observed between ATB and CTL offspring. However, plasma haptoglobin concentrations tended to be higher in HF than LF offspring, suggesting a trend for higher inflammation in HF pigs. This could reflect increased intestinal LPS entry into the body and the subsequent development of metabolic inflammation as observed in mice [2]. Data on this matter are still scarce in pigs [53], but recent investigation reported higher intestinal translocation of LPS with saturated fats than with unsaturated fats in this species [54]. We also observed a large reduction in jejunal sucrase activity in HF pigs, as already reported in pigs and rats [55], [56]. More importantly, the interaction between early ATB treatment and growing diet suggests that mechanisms of sucrase adaptation to an HF diet are altered in ATB offspring. This may be partially so for jejunal IAP (trend for an interaction).

### Conclusions and Perspectives

We developed a swine model of mild neonatal changes in microbial colonization induced by antibiotic treatment of dams around parturition. Our data show early but transient changes in intestinal enzymes and epithelial protection systems. More importantly, we disclose long-term effects of neonatal disturbances in gut colonization on intestinal function. This appears to be complex, trait-specific, site- and sometimes diet-dependent. Work is in progress to investigate the mechanisms underlying such phenotypic/functional changes deeper and to decipher the role played by gut microbiota.

## Supporting Information

Table S1 **Composition of feed.**
(DOCX)

Table S2 **Sequences of oligonucleotide primers used for real-time PCR of intestinal tissues of pigs.**
(DOCX)

Table S3 **Villus and crypt architecture of jejunal and ileal mucosa in pigs born to control or antibiotics-treated sows and fed a low (LF) or high (HF) fat diet between 140 and 169 days of age (LSmeans and SEM, n = 10 per treatment).**
(DOCX)

Table S4 **mRNA relative expression levels of heat shock proteins in ileal tissue of pigs born to control or antibiotics-treated sows and slaughtered at different ages (LSmeans and SEM, n = 9–12 per treatment).**
(DOCX)
