# Correction: Early Changes in Microbial Colonization Selectively Modulate Intestinal Enzymes, but Not Inducible Heat Shock Proteins in Young Adult Swine

**DOI:** 10.1371/journal.pone.0098730

**Published:** 2014-05-29

**Authors:** The PLOS ONE Staff

Tables 1, 2, 3, and 4, and Supporting Information Table 4 are incorrect. The author has provided corrected versions of Tables 1, 2, 3, and 4, and Table S4, which can be viewed below.

**Table 1:** Zootechnical data and plasma α-acid glycoprotein and haptoglobin concentrations in pigs born to control or antibiotics-treated sows and slaughtered at different ages (LSmeans and SEM, n  =  9-12 per treatment and age).

*Sow’s treatment Offspring’s age*	Control			Antibiotics				Statistics (P = )^ 1 ^		
	d14	d28	d42	d14	d28	d42	SEM	treat.	age	treat.*age
**Performance**										
Birth BW (kg)	1.46	1.52	1.43	1.64	1.66	1.62	0.08	0.15	0.30	0.72
Slaughter BW (kg)	4.5	8.7	14.2	4.5	8.3	13.3	0.8	0.34	<0.0001	0.63
Daily BW gain (g)	222	254	286	216	253	283	15	0.86	0.0004	0.98
**Plasma proteins of inflammation**										
α-Acid glycoprotein (µg/mL)	905	722	994	839	756	950	54	0.58	0.001	0.63
Haptoglobin (µg/mL)	579	896	1038	760	234	1262	386	0.79	0.29	0.43

1 treat.: Treatment of sows pre- and post-partum (control versus antibiotics); age (d14 and d28, unweaned; d42 weaned at d28); treat.

*age: treatment by age interaction.

**Table 2:** Morphology of the jejunum of pigs born from control or antibiotics-treated sows and slaughtered at different ages (LSmeans and SEM, n  =  9-12 per treatment and age).

*Sow’s treatment Offspring’s age*	Control			Antibiotics				Statistics (P = )^ 1 ^		
	d14	d28	d42	d14	d28	d42	SEM	treat.	age	treat.*age
**Jejunum**										
Villous height (VH, µm)	556	427	421	492	390	453	24	0.27	<0.0001	0.13
Villous width (µm)	112	147	161	115	143	163	4.6	0.96	<0.0001	0.75
Villous surface area (µm^2^, x 10^3^)	55.2	52.7	60.9	48.9	47.9	64.3	2.9	0.31	0.0004	0.22
Crypt depth (CD, µm)	194	229	403	173	277	374	26	0.97	<0.0001	0.29
Crypt width (µm)	35.6^c^	42.7^b^	52.0^a^	40.2^b^	48.0^a^	49.4^a^	1.7	0.098	<0.0001	0.051
Crypt surface area (µm^2^ x 10^3^)	6.6	8.8	18.6	6.612.6	17.0	1.3	0.49	<0.0001	0.11
VH: CD ratio	2.94	2.04	1.34	3.01	1.76	1.67	0.19	0.82	<0.0001	0.32
Absorption surface magnif. ‘M’	12.1^a^	7.6^c^	6.3^c^	10.0^b^	6.4^c^	6.9^c^	0.5	0.054	<0.0001	0.057
**Ileum**										
Villous height (VH, µm)	528	280	354	507	338	348	34	0.74	<0.0001	0.48
Villous width (µm)	113	106	151	109	115	145	4.5	0.98	<0.0001	0.16
Villous surface area (µm^2^, x 10^3^)	50.0	25.3	46.5	48.3	33.0	41.6	4.3	0.93	0.0002	0.33
Crypt depth (CD, µm)^ 2 ^	124^b^	119^b^	189^a^	135^b^	121^b^	169^a^	7	0.72	<0.0001	0.092
Crypt width (µm)44.6	43.1	56.3	44.3	45.8	53.9	1.6	0.99	<0.0001	0.28
Crypt surface area (µm^2^ x 10^3^)	4.88^c^	4.53^c^	9.19^a^	5.39^c^	4.84^c^	7.76^b^	0.45	0.59	<0.0001	0.068
VH: CD ratio	4.34	2.39	1.92	3.90	2.79	2.15	0.28	0.79	<0.0001	0.30
Absorption surface magnif. ‘M’	10.2	5.8	5.4	9.7	6.6	5.8	0.5	0.64	<0.0001	0.52

1 treat.: Treatment of sows pre- and post-partum (control versus antibiotics); age (d14 and d28, unweaned; d42 weaned at d28); treat.

*age: treatment by age interaction.

2 Crypt depth: tended to be lower in ATB than CTL group at day 42 (*P  =  0.067*).

a,b,c, Means within rows with different letters differ (P <0.05).

**Table 3:** Total Activities^1^ of aminopeptidase N, dipeptidyl-peptidase IV and sucrase in the jejunum and ileum of pigs born to control or antibiotics-treated sows and slaughtered at different ages (LSmeans and SEM, n  =  9-12 per treatment and age).

*Sow’s treatment Offspring’s age*	Control			Antibiotics				Statistics (P = )^ 1 ^		
	d14	d28	d42	d14	d28	d42	SEM	treat.	age	treat.*age
**Jejunum**										
Aminopeptidase N (APN)	10.1	10.9	10.3	9.4	8.4	8.1	1.4	0.13	0.92	0.75
Dipeptidyl-peptidase IV (DPP-IV)	0.82	0.80	0.73	0.71	0.77	1.05	0.11	0.53	0.48	0.13
Sucrase	2.9	3.2	1.2	3.0	3.0	1.6	0.6	0.83	0.014	0.88
										
**Ileum**										
Aminopeptidase N (APN)	8.3^b^	9.8^ab^	12.6^a^	6.1^b^	11.9^a^	10.2^ab^	1.1	0.38	0.0006	0.070
Dipeptidyl-peptidase IV (DPP-IV)	3.65^a^	1.78^b^	1.89^b^	2.77^ab^	3.28^a^	2.37^ab^	0.47	0.37	0.075	0.047
Sucrase (log)	-0.09	0.47	0.62	-0.41	0.50	0.55	0.11	0.20	<0.0001	0.027

1 Total activity (µmoles/min/g mucosa); x 10^-3^ for DPP-IV.

2 treat.: Treatment of sows pre- and post-partum (control versus antibiotics); age (d14 and d28, unweaned; d42 weaned at d28); treat.*age: treatment by age interaction.

a,b Means within rows with different letters differ (P <0.05).

**Table 4:** Protein expression of heat shock proteins and heat shock factor-1 in intestinal tissues of pigs born to control or antibiotics-treated sows and slaughtered at different ages (LSmeans and SEM, n  =  9-12 per treatment and age).

*Sow’s treatment Offspring’s age*	Control			Antibiotics				Statistics (P = )^ 1 ^		
	d14	d28	d42	d14	d28	d42	SEM	treat.	age	treat.*age
**Jejunum**										
HSP27/b-Actin	0.97	1.08	1.20	0.89	0.91	1.02	0.12	0.17	0.30	0.90
HSP70/b-Actin	0.61	0.74	0.81	0.52	0.59	0.63	0.07	0.031	0.12	0.84
**Ileum**										
HSP27/b-Actin	0.77	0.38	0.82	0.53	0.68	0.61	0.15	0.69	0.46	0.12
HSC70/b-Actin	0.82	0.76	0.75	1.04	1.12	0.73	0.11	0.050	0.11	0.23
HSP60/b-Actin	0.62	0.31	0.54	0.70	0.64	0.72	0.12	0.07	0.25	0.58
HSF1/b-Actin	0.33	0.38	0.41	0.33	0.31	0.41	0.08	0.69	0.51	0.87

1 treat.: Treatment of sows pre- and post-partum (control versus antibiotics); age (d14 and d28, unweaned; d42 weaned at d28); treat.

*age: treatment by age interaction.

## Supporting Information

Table S4 mRNA relative expression levels of heat shock proteins in ileal tissue of pigs born to control or antibiotics-treated sows and slaughtered at different ages (LSmeans and SEM, n  =  9–12 per treatment).
(DOCX)
