# Supplementary material for: Early Changes in Microbial Colonization Selectively Modulate Intestinal Enzymes, but Not Inducible Heat Shock Proteins in Young Adult Swine
Source: PLoS One. 2014 Feb 4;9(2):e87967. doi: 10.1371/journal.pone.0087967 (PMC3913709; doi:10.1371/journal.pone.0087967)
Supplement: Table S1 — Composition of feed. (DOCX) [file pone.0087967.s001.docx]

**Table S1.** Composition of feed.

| **Feed^1^** | **PS** | **ST** | **GR** | **GE** | **LA** |
| --- | --- | --- | --- | --- | --- |
| **Composition, %** |  |  |  |  |  |
| Wheat |  | 23.2 | 26.2 | 22.0 | 25.6 |
| Corn |  | 25.0 | 16.0 | 10.0 | 12.0 |
| Barley | 45.3 | 24.1 | 25.5 | 33.9 | 25.7 |
| Wheat bran |  |  | 5.0 | 15.0 | 10.0 |
| Soybean meal | 17.5 | 22.6 | 19.0 | 9.0 | 18.0 |
| Soybean protein | 2.5 |  |  |  |  |
| Vegetal oil | 2.3 | 0.5 | 2.0 | 2.0 | 2.0 |
| Molasses |  |  | 3.0 |  | 3.0 |
| Sugar beet pulp |  |  |  | 5.0 |  |
| Sweet whey | 20.0 |  |  |  |  |
| Refatted (40%) skimmed milk powder | 8.0 |  |  |  |  |
| Calcium carbonate | 1.41 | 1.13 | 1.29 | 1.74 | 1.20 |
| Monocalcium phosphate | 0.80 | 0.97 |  |  |  |
| Dicalcium phosphate |  |  | 0.50 | 0.30 | 1.02 |
| Salt |  | 0.40 | 0.45 | 0.45 | 0.45 |
| Trace elements and vitamin premix | 0.50 | 0.50 | 0.50 | 0.50 | 0.50 |
| Amino acids, acidifier and phytase^2^ | 1.69 | 1.60 | 0.56 | 0.11 | 0.53 |
|  |  |  |  |  |  |
| **Chemical composition, %** |  |  |  |  |  |
| Minerals | 7.0 | 5.4 | 5.6 | 5.8 | 6.1 |
| Crude protein | 19.0 | 18.0 | 16.5 | 13.3 | 16.4 |
| Crude fat | 6.7 | 2.8 | 4.2 | 4.3 | 4.2 |
| Crude fiber | 3.0 | 3.6 | 3.8 | 5.1 | 4.1 |
| Starch | 24.5 | 43.5 | 40.9 | 40.5 | 38.9 |
|  |  |  |  |  |  |
| **Nutritional value (Mcal or g/kg)** |  |  |  |  |  |
| Digestible energy | 3.48 | 3.24 | 3.21 | 3.06 | 3.14 |
| Net energy | 2.54 | 2.31 | 2.31 | 2.21 | 2.25 |
| Digestible lysine | 12.8 | 11.4 | 8.4 | 4.8 | 8.4 |
| Digestible methionine + cysteine | 7.8 | 6.8 | 5.2 | 4.1 | 5.2 |
| Digestible threonine | 8.1 | 7.1 | 5.2 | 3.6 | 5.2 |
| Digestible tryptophan | 2.5 | 2.2 | 1.7 | 1.3 | 1.7 |
| Digestible phosphorus | 4.1 | 3.9 | 2.7 | 2.4 | 3.5 |
| Calcium | 12.5 | 9.5 | 10.0 | 11.5 | 11.0 |

^1^Manufacturer: Cooperl, Lamballe, France. Feed: PS=Pre-starter; ST=Starter; GR=growing; GE = gestating; LA = Lactating.

^2^Detailed supplementation not disclosed by the manufacturer.

PS, ST, GR and LA feed supplemented with lysine, methionine and threonine; PS and ST supplemented with tryptophan and valine; All feed supplemented with acidifier (anti-mold) and phytase.
