# Supplementary material for: Early Changes in Microbial Colonization Selectively Modulate Intestinal Enzymes, but Not Inducible Heat Shock Proteins in Young Adult Swine
Source: PLoS One. 2014 Feb 4;9(2):e87967. doi: 10.1371/journal.pone.0087967 (PMC3913709; doi:10.1371/journal.pone.0087967)
Supplement: Table S2 — Sequences of oligonucleotide primers used for real-time PCR of intestinal tissues of pigs. (DOCX) [file pone.0087967.s002.docx]

**Table S2.** Sequences of oligonucleotide primers used for real-time PCR of intestinal tissues of pigs.

| **Gene** |  | **Primer** | Accession number | **Reference** |
| --- | --- | --- | --- | --- |
| CycloA | Forward | 5’-TAACCCCACCGTCTTCTT-3’ | F14571, EMBL | [30] |
|  | Reverse | 5’-TGCCATCCAACCACTCAG-3’ |  |  |
| IAP | Forward | 5’-CACCTGTCTGTCCACGTTGT-3’ | AY 145131, NCBI | [31] |
|  | Reverse | 5’-CTAAAGGGGCAGATGAATGG-3’ |  |  |
| APN | Forward | 5’-GGTACACGTCGCCTGGCACG-3’ | Z 29522, NCBI | [31] |
|  | Reverse | 5’-TGACCACGCAGAGCCCACCT-3’ |  |  |
| Sucrase^1^ | Forward | 5’-ACTCTTGCCATAAGCAGCCACGT-3’ | NM_001041, NCBI; NM_013061, NCBI | [31] |
|  | Reverse | 5’-TGGGATCAAACTTTCTTGGACGCTG-3’ |  |  |
| DPPIV^2^ | Forward | 5’-TGGATTCAGCTCACAGCT-3’ | X73277, NCBI | [32] |
|  | Reverse | 5’-CCTCCGGCGTCTGTGTTA-3’ |  |  |
| HSP27 | Forward | 5’-CCGGTGTTTCACTCGAAAATACA-3’ | NM_001007518, NCBI | [33] |
|  | Reverse | 5’-GCTTTTCCGACTTTCCAGCTTCT-3’ |  |  |
| HSP70 | Forward | 5’-GCCCTGAATCCGCAGAATA-3’ | X68213, NCBI | [34] |
|  | Reverse | 5’-TCCCCACGGTAGGAAACG-3' |  |  |

^1^ Oligonucleotides to recognize pig sucrase were designed from regions of high homology between sucrase cDNA sequences from human (NM_001041) and rat (NM_013061).

^2^ RT-PCR for DPPIV did not work with these primers in the present study.
