# Supplementary material for: Early Changes in Microbial Colonization Selectively Modulate Intestinal Enzymes, but Not Inducible Heat Shock Proteins in Young Adult Swine
Source: PLoS One. 2014 Feb 4;9(2):e87967. doi: 10.1371/journal.pone.0087967 (PMC3913709; doi:10.1371/journal.pone.0087967)
Supplement: Table S3 — Villus and crypt architecture of jejunal and ileal mucosa in pigs born to control or antibiotics-treated sows and fed a low (LF) or high (HF) fat diet between 140 and 169 days of age (LSmeans and SEM, n = 10 per treatment). (DOCX) [file pone.0087967.s003.docx]

**Table S3.** Villus and crypt architecture of jejunal and ileal mucosa in pigs born to control or antibiotics-treated sows and fed a low (LF) or high (HF) fat diet between 140 and 169 days of age (LSmeans and SEM, n = 10 per treatment).

| *Sow’s treatment* | **Control** | |  | **Antibiotics** | |  |  |  | **Statistics (P =)^1^** | |  |
| --- | --- | --- | --- | --- | --- | --- | --- | --- | --- | --- | --- |
| *Offspring’s diet* | **LF** | **HF** |  | **LF** | **HF** |  | **SEM** |  | **treat.** | **diet** | **treat.*diet** |
| **Jejunum** |  |  |  |  |  |  |  |  |  |  |  |
| Villus height (µm) | 567 | 581 |  | 513 | 582 |  | 33 |  | 0.45 | 0.21 | 0.41 |
| Villus width (µm) | 171 | 172 |  | 179 | 174 |  | 7 |  | 0.56 | 0.77 | 0.68 |
| Villus surface area (µm² x 10^3^) | 883 | 91.5 |  | 79.9 | 91.2 |  | 5.6 |  | 0.47 | 0.20 | 0.46 |
| Crypt depth (µm) | 504 | 501 |  | 501 | 509 |  | 22 |  | 0.90 | 0.91 | 0.81 |
| Crypt width (µm) | 44 | 43 |  | 47 | 45 |  | 1 |  | 0.029 | 0.22 | 0.77 |
| Crypt surface area (µm² x 10^3^) | 22.1 | 22.3 |  | 23.1 | 22.1 |  | 1.1 |  | 0.46 | 0.42 | 0.93 |
| VH: CD ratio | 1.22 | 1.21 |  | 1.02 | 1.19 |  | 0.10 |  | 0.33 | 0.45 | 0.38 |
| Absorption surface magnif. ‘M’ | 8.8 | 9.1 |  | 7.6 | 8.9 |  | 0.6 |  | 0.27 | 0.18 | 0.40 |
|  |  |  |  |  |  |  |  |  |  |  |  |
| **Ileum** |  |  |  |  |  |  |  |  |  |  |  |
| Villus height (µm) | 443 | 422 |  | 425 | 416 |  | 21 |  | 0.58 | 0.46 | 0.75 |
| Villus width (µm) | 208 | 195 |  | 205 | 205 |  | 8 |  | 0.66 | 0.38 | 0.33 |
| Villus surface area (µm² x 10^3^) | 79.8 | 73.2 |  | 77.3 | 75.0 |  | 4.6 |  | 0.95 | 0.33 | 0.63 |
| Crypt depth (µm) | 380 | 390 |  | 407 | 389 |  | 13 |  | 0.35 | 0.78 | 0.29 |
| Crypt width (µm) | 50.0 | 49.6 |  | 46.1 | 48.1 |  | 1.6 |  | 0.11 | 0.62 | 0.45 |
| Crypt surface area (µm² x 10^3^) | 18.8 | 19.3 |  | 18.5 | 18.5 |  | 0.8 |  | 0.52 | 0.78 | 0.76 |
| VH: CD ratio | 1.19 | 1.09 |  | 1.05 | 1.07 |  | 0.07 |  | 0.30 | 0.57 | 0.42 |
| Absorption surface magnif. ‘M’ | 6.49 | 6.47 |  | 6.51 | 6.28 |  | 0.28 |  | 0.77 | 0.64 | 0.70 |

**^1^** treat.: Treatment of sows pre- and post-partum (control versus antibiotics); diet (low versus high fat diet); treat.*diet: treatment by diet interaction.
