# Supplementary material for: Correction: Early Changes in Microbial Colonization Selectively Modulate Intestinal Enzymes, but Not Inducible Heat Shock Proteins in Young Adult Swine
Source: PLoS One. 2014 May 29;9(5):e98730. doi: 10.1371/journal.pone.0098730 (PMC4038587; doi:10.1371/journal.pone.0098730)
Supplement: Table S4 — mRNA relative expression levels of heat shock proteins in ileal tissue of pigs born to control or antibiotics-treated sows and slaughtered at different ages (LSmeans and SEM, n = 9–12 per treatment). (DOCX) [file pone.0098730.s001.docx]

**Table S4.** mRNA relative expression levels of heat shock proteins in ileal tissue of pigs born to control or antibiotics-treated sows and slaughtered at different ages (LSmeans and SEM, n = 9-12 per treatment).

| *Sow’s treatment* | **Control** | | |  | **Antibiotics** | | |  |  |  | **Statistics (P =)^1^** | |  |
| --- | --- | --- | --- | --- | --- | --- | --- | --- | --- | --- | --- | --- | --- |
| *Offspring’s age* | **d14** | **d28** | **d42** |  | **d14** | **d28** | **d42** |  | **SEM** |  | **treat.** | **age** | **treat.*age** |
| hsp27/gapdh | 1.03 | 0.94 | 0.97 |  | 1.00 | 0.91 | 0.91 |  | 0.12 |  | 0.70 | 0.73 | 0.99 |
| hsp27/cycloA | 1.05 | 0.94 | 0.99 |  | 1.00 | 0.91 | 0.91 |  | 0.15 |  | 0.70 | 0.78 | 0.98 |
| hsp70/gapdh | 1.04 | 0.87 | 1.05 |  | 1.00 | 0.19 | 0.84 |  | 0.28 |  | 0.20 | 0.18 | 0.50 |
| hsp70/cycloA | 1.07 | 0.85 | 1.09 |  | 1.00 | 0.27 | 0.85 |  | 0.31 |  | 0.27 | 0.27 | 0.70 |

**^1^** treat.: Treatment of sows pre- and post-partum (control versus antibiotics); age (d14 and d28, unweaned; d42 weaned from d28); treatment by age interaction.
